# Bilateral borderline ovarian Brenner tumor coexisting with low-grade appendiceal mucinous neoplasm in a perimenopausal woman: a case report

**DOI:** 10.11604/pamj.2025.52.68.49272

**Published:** 2025-10-13

**Authors:** Fang Zhang, Minjie Liu, Jingfei Zheng

**Affiliations:** 1 Department of Gynecology, The Affiliated People’s Hospital of Ningbo University, Ningbo, Zhejiang, China

**Keywords:** Borderline Brenner tumor, low-grade appendiceal mucinous neoplasm, bilateral ovarian neoplasm, laparoscopic surgery, case report

## Abstract

Borderline ovarian Brenner tumor (BOT-B) accounts for less than 2% of all ovarian neoplasms. It is typically unilateral and large, with non-specific imaging features. Synchronous extra-ovarian neoplasia is rarely documented. A 47-year-old gravida 2, para 2 woman presented with intermenstrual bleeding and lower abdominal discomfort. Transvaginal ultrasound and pelvic magnetic resonance imaging (MRI) revealed a 9 cm right adnexal complex mass without ascites or elevated serum tumor markers. Intraoperative findings identified bilateral, firm, lobulated ovarian tumors (right: 5 cm; left: 7 cm) and an inflamed, hydropic appendix. Frozen section analysis of the left ovary indicated a borderline Brenner tumor with focal atypia; carcinoma could not be ruled out. Following informed consent, the patient underwent laparoscopic bilateral salpingo-oophorectomy, appendicectomy, omentectomy, and peritoneal biopsy. Final histopathology confirmed bilateral BOT-B without stromal invasion and a synchronous low-grade appendiceal mucinous neoplasm (LAMN) with negative margins. No adjuvant chemotherapy was administered. Imaging at three-month follow-up showed no evidence of recurrence. This represents the first reported case of synchronous bilateral BOT-B and LAMN. It highlights the following: i) the critical importance of comprehensive surgical exploration for atypical adnexal masses; ii) that BOT-B can present bilaterally and at a smaller size than classically described; and iii) that complete, conservative fertility-sparing surgery with strict adherence to a tumor-free technique is an adequate management strategy for borderline Brenner tumors, associated with excellent short-term outcomes.

## Introduction

Borderline ovarian Brenner tumor (BOT-B) is a rare entity, accounting for less than 2% of all ovarian neoplasms [1,2]. The overwhelming majority are unilateral, measure more than 10 cm, and are discovered incidentally in postmenopausal women; bilateral involvement is reported in only 5-14% of cases [3]. Precise preoperative diagnosis is unattainable, as imaging lacks pathognomonic features, and diagnosis ultimately depends on histopathological verification [4]. Consequently, each new case that deviates from the classic presentation, particularly in laterality, size, or co-existing pathology, expands the limited evidence base necessary for refining diagnostic algorithms and surgical strategies. The present case is unique for three principal reasons. First, it documents bilateral BOT-B in a perimenopausal patient with a combined ovarian diameter of less than 10 cm, a rarely reported clinical scenario [3]. Second, a concomitant low-grade appendiceal mucinous neoplasm (LAMN) was identified unexpectedly during the same procedure; to our knowledge, this represents the first report of synchronous BOT-B and LAMN. Third, complete cytoreduction was achieved laparoscopically without hysterectomy, confirming that fertility-sparing, image-guided surgery with frozen-section support can be both safe and curative for carefully selected patients with borderline Brenner tumors. This report therefore contributes significant new insights to the literature concerning the epidemiology, synchronous pathology, and minimally invasive management of these rare neoplasms.

## Patient and observation

**Patient information:** a 47-year-old gravida 2, para 2 woman (coded ID: BBL-001) presented with a single episode of intermenstrual spotting and two days of lower abdominal heaviness. A transvaginal ultrasound revealed a 9 cm right adnexal mass. Her medical history included hypertension for eight years, for which she was irregularly medicated. She had no history of diabetes, prior surgery, malignancy, or familial cancer. Menarche occurred at age 13, and her current menstrual cycle intervals had shortened to 24 days. A follicle-stimulating hormone (FSH) level of 21.4 IU/L suggested perimenopause. No genetic testing had been performed. Two years prior, a 4 cm simple right ovarian cyst had been documented but was lost to follow-up. The current mass represented her first indication for operative intervention.

**Clinical findings:** on admission, the patient's vital signs were stable, with a blood pressure of 138/86 mmHg, a heart rate of 78 bpm, and she was afebrile. Abdominal examination revealed mild suprapubic tenderness without guarding or a palpable mass. Upon pelvic examination, the uterus was normal-sized and mobile. A large, 8x10cm, cystic-solid, non-tender, and freely mobile right adnexal mass was identified, which extended into the anterior cul-de-sac. The left adnexa were clear, and a rectovaginal examination was unremarkable. Laboratory investigations demonstrated normal tumor marker levels, including CA-125, CA-19-9, CEA, AFP, and SCC antigen. A β-hCG test was negative, and an FSH level of 21.4 IU/L was consistent with perimenopause.

**Timeline of the current episode:** the patient was referred and admitted to our unit on May 5, 2025, following an episode of intermenstrual bleeding and a two-day history of lower abdominal heaviness, which prompted an urgent workup. Diagnostic laparoscopy with bilateral salpingo-oophorectomy, appendectomy, omentectomy, and peritoneal biopsies was performed on May 7^th^, 2025. The final pathology report, issued on May 13^th^, 2025, confirmed a bilateral borderline Brenner tumor and an incidental low-grade appendiceal mucinous neoplasm. The patient was discharged without complication on May 14^th^, 2025. Post-discharge follow-up reviews, including clinical examination and pelvic ultrasound, were conducted on June 14^th^, 2025 and August 15^th^, 2025; all showed no evidence of recurrence or residual disease.

**Figure 1:**
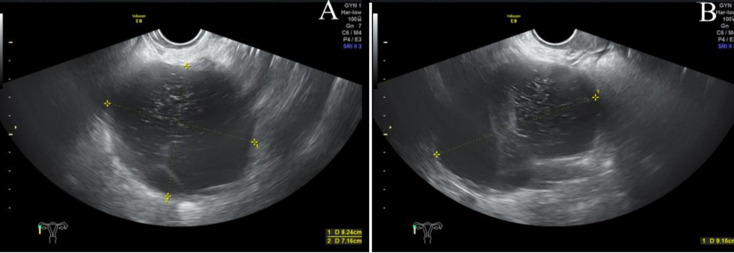
transvaginal 3D Ultrasound Imaging of the uterine adnexa; A) a well-defined, anechoic cystic mass (82x72 mm in transverse and anteroposterior dimensions) in the right adnexa; B) the same right adnexal mass, measuring 92 mm in length, with internal septations and fine echogenic spots

**Figure 2:**
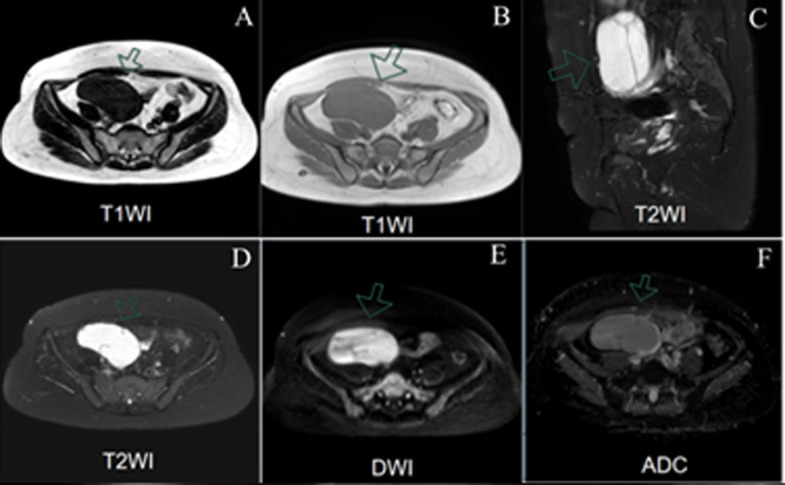
pelvic MRI findings of a right adnexal mass; A, B) T1-weighted imaging (T1WI) showing a well-defined mass with smooth margins and homogeneous intermediate signal intensity in the right adnexa; C, D) T2-weighted imaging (T2WI) reveals a hyperintense mass containing hypointense septations; E, F) Diffusion-weighted imaging (DWI) showing high signal intensity with slightly reduced ADC values, indicating mildly restricted diffusion

**Figure 3:**
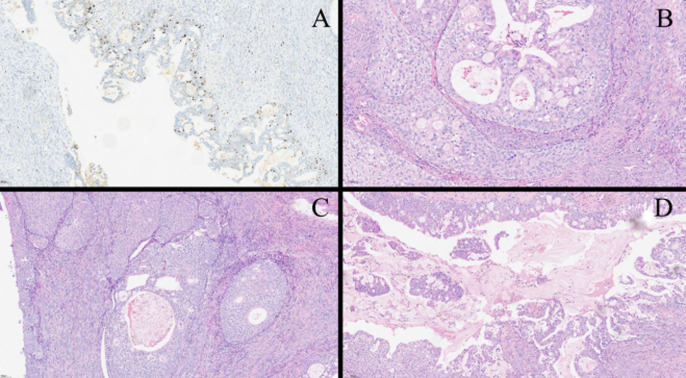
histopathological findings of the resected specimen (H&E staining); A) Ki-67 immunostaining (10X): increased Ki-67 proliferation index, up to 20% in hot spots; B) mitotic figures (20X, blue arrows): tumor cells are crowded and mitotically active; C) benign Brenner tumor (10X): nests of transitional epithelium with cystic change within a fibromatous stroma; uniform cells with pale cytoplasm and small nucleoli; D) papillary structures (10X): crowded epithelium with papillae; cytologic atypia and mitoses are present without stromal invasion

**Diagnostic assessment:** the diagnostic workup revealed normal tumor markers (CA-125, CA-19-9, CEA, AFP, SCC) and a negative β-hCG. A follicle-stimulating hormone (FSH) level of 21.4 IU/L was consistent with perimenopause. Transvaginal ultrasound identified a 92x82x72mm right adnexal mass with cystic-solid and septated features (Figure 1). Subsequent pelvic MRI demonstrated a T2-high signal intensity lesion with low-signal septa and restricted diffusion, raising concern for a cystadenoma or cystadenocarcinoma (Figure 2). No ascites or lymphadenopathy was present. Financial constraints had previously precluded follow-up of a known 4 cm cyst, resulting in a diagnostic gap. An intraoperative frozen section was obtained from the left ovarian tumor, and its analysis suggested a borderline Brenner tumor with focal atypia, noting that malignancy could not be excluded. Final histopathology established a definitive diagnosis of bilateral borderline ovarian Brenner tumors without stromal invasion (Stage IA) and an incidental, completely excised low-grade appendiceal mucinous neoplasm (Figure 3). Given the excellent prognosis of both entities with surgical management alone, no adjuvant therapy was indicated.

**Diagnosis:** the final diagnosis was bilateral borderline ovarian Brenner tumors (International Federation of Gynecology and Obstetrics (FIGO) Stage IA) and a concurrent, completely resected low-grade appendiceal mucinous neoplasm.

**Figure 4:**
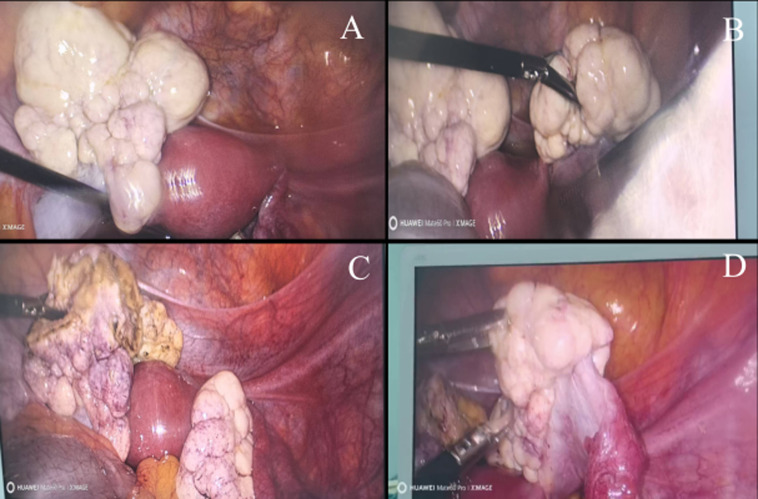
laparoscopic intraoperative findings; A) the left ovary (above the grasping forceps) appears whitish and lobulated; B) bilateral ovaries are enlarged with a whitish, lobulated morphology; C) the resected left ovary is held by grasping forceps; D) the right ovary is grasped prior to excision

**Therapeutic interventions:** during the procedure, systematic exploration revealed no abnormalities in the pelvic peritoneum, upper abdominal organs (liver, gallbladder, spleen, stomach), or intestinal tract. The uterus appeared normal. The left ovary contained a firm, lobulated 5x6x7cm mass. Due to elongation of the left ovarian ligament, the tumor was displaced into the right Douglas pouch and adhered to the right ovary. The right ovary contained a similar firm, lobulated 4x4x5cm mass with a grayish-white surface. The appendix appeared tortuously elongated with edematous, thickened, and suppurative changes at its tip. Following intraoperative consultation with gastrointestinal surgeons, appendectomy was indicated. Frozen-section analysis of the left ovarian tumor suggested borderline Brenner tumor with focal atypia, though malignancy could not be entirely excluded. Definitive surgical management was completed laparoscopically in a single session under general anesthesia. The procedure entailed bilateral salpingo-oophorectomy, appendectomy, omentectomy, and systematic peritoneal biopsies, achieving complete cytoreduction with no residual disease (Figure 4). As both the bilateral borderline Brenner tumors (Stage IA) and the incidental low-grade appendiceal mucinous neoplasm were completely excised and carry an excellent prognosis with surgery alone, no adjuvant chemotherapy, hormonal therapy, or targeted agents were administered. The original surgical plan, upheld after frozen-section confirmation of clear margins and absence of invasive disease, obviated the need for hysterectomy or lymphadenectomy, thereby preserving the uterus for potential future hormone replacement. Postoperative care included standard thromboprophylaxis (enoxaparin 40 mg subcutaneously daily for 7 days) and early mobilization. The patient recovered without complications and was discharged on postoperative day 9.

**Follow-up and outcomes:** the patient reported complete resolution of abdominal discomfort and a return to regular activity at 7, 14, and 30 days post-discharge. Clinical assessment during these intervals confirmed an Eastern Cooperative Oncology Group (ECOG) performance status of 0 and a visual analogue scale (VAS) pain score of 0/10. A gynecological examination and transvaginal ultrasound at three months revealed no residual mass, ascites, or pelvic lymphadenopathy. Serum tumor markers (CA-125, CA-19-9, and CEA) remained within normal limits, confirming continued remission. The patient adhered to thromboprophylaxis with self-administered enoxaparin and early mobilization instructions, as documented by nursing telephone calls on postoperative days 7 and 14; no medication-related bruising or wound complications were reported. No adverse events, such as infection, port-site hernia, bowel injury, or recurrent bleeding, occurred during the 90-day follow-up period.

**Patient perspective:** the patient was delighted with the quality of care.

**Informed consent:** written informed consent was obtained from the patient for the publication of this case report.

## Discussion

The strengths of this report comprise the first description of synchronous bilateral borderline ovarian Brenner tumors (BOT-B) and a low-grade appendiceal mucinous neoplasm (LAMN), their complete laparoscopic resection with negative margins, and 90-day imaging and biochemical documentation of remission. Limitations include the absence of germline or somatic mutational profiling and a follow-up duration shorter than the five-year interval recommended for rare ovarian neoplasms. Borderline ovarian Brenner tumors constitute less than 2% of ovarian tumors; bilateral presentation is cited in only 5-14% of cases, and tumors typically exceed 10 cm in diameter. In contrast, our patient was perimenopausal with a combined ovarian diameter of less than 10 cm [1,3]. Imaging lacks pathognomonic features; while low-signal septa on T2-weighted MRI and fine dotted echoes and septations on ultrasound are suggestive, they are not diagnostic [4]. Consequently, intraoperative frozen section analysis is critical. The finding of transitional-cell nests with moderate atypia, but no stromal invasion, correctly guided the decision against comprehensive staging while confirming complete cytoreduction.

Synchronous LAMN has not been previously reported with BOT-B. Both lesions are thought to originate from transitional or urological-type epithelium and share an indolent natural history when completely excised [5]. As neither entity benefits from adjuvant chemotherapy, fertility-sparing surgery that adheres to oncological principles is justified in women approaching menopause who decline hysterectomy. Our patient´s intact uterus preserves future hormone-replacement options and does not compromise oncological safety. The key lesson is that bilateral, small-volume BOT-B can coexist with an incidental LAMN, underscoring the necessity for systematic abdominal exploration and appendix assessment during adnexal surgery. Laparoscopic bilateral salpingo-oophorectomy plus appendectomy with omental and peritoneal sampling achieved complete remission without staging lymphadenectomy or chemotherapy, supporting a conservative yet thorough surgical approach for carefully selected borderline Brenner tumors.

## Conclusion

This first report of concurrent bilateral borderline ovarian Brenner tumors and a low-grade appendiceal mucinous neoplasm demonstrates that even small-volume, imaging-nonspecific adnexal masses warrant systematic abdominal exploration. Complete laparoscopic resection without hysterectomy or lymphadenectomy resulted in disease-free status at 3-month follow-up, supporting a conservative yet thorough surgical approach for carefully selected patients with borderline Brenner tumors.
